# Effect of Music on Anxiety and Fatigue in Cancer Patients Undergoing Chemotherapy: A Randomized Controlled Trial

**DOI:** 10.34172/aim.31258

**Published:** 2024-11-01

**Authors:** Havva Gezgin Yazıcı, Çiğdem Ökten, Esra Karabulut, Mehmet Aliustaoğlu

**Affiliations:** ^1^Mental Health Nursing Department, Faculty of Health Sciences, Kütahya Health Sciences University, Kütahya, Turkey; ^2^Internal Medicine Nursing Department, Faculty of Health Sciences, Kütahya Health Sciences University, Kütahya, Turkey; ^3^Internal Medical Sciences, School of Medicine, Kütahya Health Sciences University, Kütahya, Turkey

**Keywords:** Anxiety, Cancer, Chemotherapy, Fatigue, Nursing, Music intervention

## Abstract

**Background:**

Anxiety and fatigue are symptoms typically experienced by cancer patients undergoing chemotherapy. In dealing with these symptoms, listening to music may help patients.

**Methods:**

The randomized controlled study was conducted between May 1, 2022 and November 10, 2022 with 60 patients treated in the outpatient chemotherapy unit. The data were gathered using a patient information form, Brief Fatigue Inventory, and the State Trait Anxiety Inventory. In addition to standard treatment and care, 30-minute music was played with a passive listening method in a total of three cycles of chemotherapy in the intervention group patients who completed the first cycle and visited for the second cycle of chemotherapy.

**Results:**

According to the analysis of covariance (ANCOVA) findings, the group variable significantly affected the post-test state anxiety scores when an adjustment was made for pre-test state anxiety scores (F=240.398, *P*<0.001, η^2^=0.808). In addition, pre-test state anxiety scores affected post-test results (F=7.925, *P*=0.007, η^2^=0.122). According to the ANCOVA findings, the group variable significantly affected the post-test trait anxiety scores (F=235.243, *P*<0.001, η^2^=0.805). In addition, pre-test trait anxiety scores affected post-test results (F=34.977, *P*<0.001, η^2^=0.380). According to ANCOVA results, the group variable significantly affected post-test fatigue scores (F=79.201, *P*<0.001, η^2^=0.582). In addition, pre-test scores affected post-test scores (F=11.082, *P*=0.002, η^2^=0.163).

**Conclusion:**

We observed that music had positive effects on fatigue and anxiety levels in cancer patients undergoing chemotherapy. It may be recommended to include music intervention in nursing practices for cancer patients during chemotherapy. The study results demonstrated that music intervention can be used in nursing practices for cancer patients during chemotherapy. Its low-cost and non-invasive nature also provide ease of application. Therefore, we can recommend the application of music intervention in outpatient chemotherapy units.

## Introduction

 Chemotherapy, which is among the standard treatments for cancer, has various side effects that cause a significant decrease in quality of life. Severe side effects may result in dose reduction or discontinuation of treatment. Therefore, early interventions that can enhance treatment tolerance can be very crucial in increasing recovery chances.^1^ Today, music is used in various fields in the treatment and rehabilitation of diseases. Music interventions are known for their comfort, non-invasiveness, and high practicability and can be categorized into two categories: music medicine (implemented by medical or health professionals) and music therapy (implemented by specialized music therapists).^2^ The effectiveness of music medicine mainly involves the use of music itself directly on diseases and symptoms.^3^ The music medicine approach is very valuable in terms of benefiting from the positive impacts of music on the physiological and psychological parameters of patients whose health status is not suitable for participating in active music therapy practices.^3^ Listening to music can facilitate relaxation in the patient by positively affecting neurophysiological and emotional responses.^4^ Music interventions can help to cope with the intense feelings and stress that occur in chemotherapy sessions by stimulating the cortical region of the brain.^5^

 Music interventions stimulate functional processes in the brain, such as stimulation of endorphins, dopamine, and serotonin and rapid activation of neural pathways. Hence, positive and pleasing feelings increase and the severity of fatigue decreases.^2^ Music interventions aimed at reducing the severity of fatigue provide high practicability, arouse positive feelings, and ensure the well-being of patients.^6^ In a music therapy study conducted during the treatment of adult cancer patients, it was found that the chemotherapy and radiotherapy-related perception of pain, nausea, vomiting, fatigue, and stress associated with physical discomfort decreased.^7^ Li et al reported that music interventions were effective in improving the quality of life and reducing pain, depression, and anxiety in cancer patients.^8^ Another study on breast cancer patients found that music was similarly effective in reducing anxiety.^1^

 In a meta-analysis, it was found that music interventions were effective in significantly improving pain, anxiety, depression, and fatigue in cancer patients, especially adults.^9^ When music interventions in oncology are examined, it is seen that it has positive effects on physiological parameters as well as anxiety, depression, fatigue and pain in cancer patients.^10^

 This study aimed to examine the effect of music on fatigue and anxiety in cancer patients undergoing chemotherapy. The hypotheses of the study were:

a) Music intervention has an effect on reducing fatigue in cancer patients undergoing chemotherapy.
b) Music intervention has an effect on reducing state-trait anxiety in cancer patients undergoing chemotherapy.

## Materials and Methods

###  Study Design

**Figure 1:**
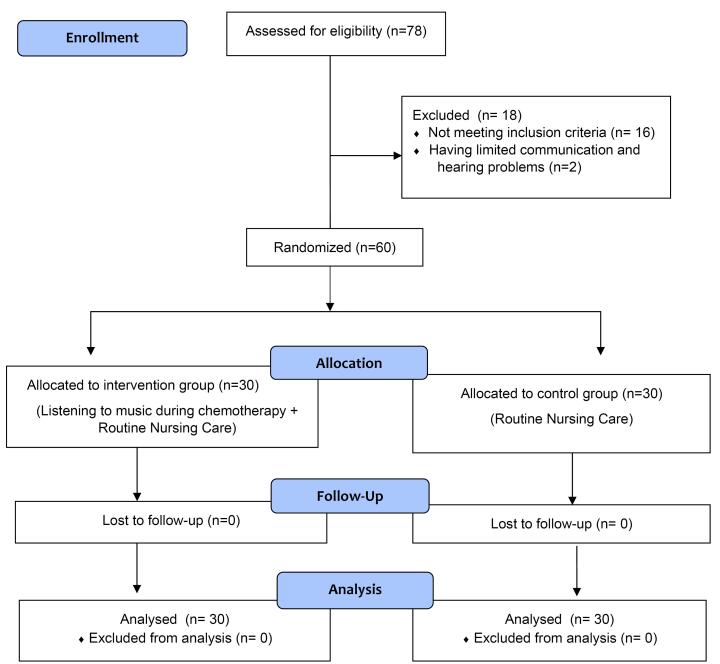
CONSORT Diagram Showing Participant Flow Through the Study

 This is a single-blind experimental study with randomized control. Patients who agreed to participate in the study and met the inclusion criteria were randomly assigned to either the control group (patients who were given standard treatment and care) or intervention group (patients who received music intervention). Figure 1 shows the CONSORT (Consolidated Standards of Reporting Trials) flow chart of this study.^11^

###  Participants

 The research was conducted between May 1, 2022 and November 10, 2022 in an outpatient chemotherapy unit of a training and research hospital. The population of the study consisted of 78 patients diagnosed with cancer who received treatment in the outpatient chemotherapy unit of this training and research hospital. A power analysis was conducted to determine the number of participants required for the study. The power of the test was calculated using the G*Power 3.1 program. In a similar study conducted by Lin and colleagues in the relevant literature, the effect size for anxiety change was calculated as 0.861. To ensure that the power of the study exceeds 99%, it was necessary to recruit 54 participants in total, with 27 participants in each group, at a significance level of 5% and an effect size of 0.861 (df = 26; t = 2.056). Considering potential losses and to achieve a high power in the test, the target was set to 60 participants in total, with 30 participants in each group. To avoid bias in the selection of participants, randomization was performed.^12^ The inclusion criteria were as follows (1) being diagnosed with cancer, (2) undergoing outpatient chemotherapy treatment, (3) being 18 years of age or older, (4) ability to speak, read, and write in Turkish, (5) agreeing to participate in the study, and (6) in order to work during the treatment periods when the side effects of chemotherapy started to be intensively observed, we selected patients who completed the first cycle of chemotherapy and who were undergoing the second cycle of chemotherapy. The exclusion criteria included (1) those diagnosed with psychiatric diseases and (2) Those with limited communication and hearing problems.

###  Randomization and Blinding

 The randomization process of the sample into an intervention and a control group was carried out in consultation with a statistical expert. A simple randomization method was used. The Randomizer.org program was used to determine the patients in the intervention and control groups. In the randomization process, single blinding was performed where the participants did not know to which group (intervention group/control group) they were assigned.

## Procedures

 In this study, with an intervention and control group, the intervention group listened to 30 minutes of music with the passive listening method in addition to standard treatment and care in three cycles of chemotherapy, and only standard treatment and care were applied to the control group. Before the music intervention, a 10-question Patient Information Form, the Short Form Fatigue Scale (BFI), and the State - Trait Anxiety Inventory (STAI) were applied to both groups. While the patients in the intervention group were undergoing chemotherapy, the instrumental music pieces in the Rast tonality were listened to with the passive listening method in addition to routine treatment. These works were obtained from the page of TÜMATA (The Group for the Research and Promotion of Turkish Music). The reason for the selection of the Rast tonality is that it gives joy, peace of mind, and comfort to the person.^13^ No application besides routine treatment and care was applied to the control group, and after the intervention group listened to music, the BFI and the STAI were re-applied to the intervention and control groups.

###  Data Collection

 The data were collected between May 1, 2022 and November 10, 2022. A Patient Information Form was applied to both groups at the beginning of the study. Pre-test (Time 1) was applied to both groups before music intervention (patients who visited for the 2^nd^ cycle chemotherapy treatment), and post-test (Time 2) was applied to both groups at the end of the 4^th^ cycle chemotherapy treatment after the intervention group listened to music during three cycles of chemotherapy.

###  Instruments

####  Patient Information Form

 The form, consisting of 10 questions and prepared by the researchers, contains information about the individuals’ sociodemographic characteristics and disease.^1,3,13-16^

####  Brief Fatigue Inventory (BFI)

 It is one of the standardized tests used to evaluate fatigue in cancer patients. The Turkish validity of the test developed by MD Anderson Cancer Center was assessed by Çınar et al.^17^ The nine-item scale examines the fatigue level in the last 24 hours and the reflection of this fatigue on activities of daily living.^18^ It was stated that the BFI was suitable for the Turkish society. The Cronbach’s alpha of the inventory was found to be 0.98. The Cronbach’s alpha of the inventory in our study was found to be 0.98.

####  State Trait Anxiety Inventory (STAI)

 It was developed by Spielberg et al in 1970 and its reliability and validity for Turkey were assessed by Öner and Le Compte. The scale is a type of self-assessment scale with short expressions. It consists of two subscales, each containing 20 expressions and measuring state and trait anxiety separately. The score of the scales varies between 20‒80. The Cronbach’s alpha of the scale was found to be between 0.83 and 0.92 for the state anxiety scale and between 0.83 and 0.87 for the trait anxiety scale.^19^ The Cronbach’s alpha values of the scale in our study were found to be 0.60 and 0.72.

###  Data Analysis

 The SPSS v23 statistical program was used in the analysis of the study. Demographic characteristics were assessed by chi-square test. Descriptive statistics (mean, standard deviation) were used to define the main variables. All variables were normally distributed; therefore, parametric tests (analysis of covariance [ANCOVA] and independent *t* test) were used to determine the differences in variables between groups. A two-sided probability value *<*0.05 was considered to be statistically significant. Kurtosis and skewness values were evaluated to determine whether the research variables complied with normal distribution. In the literature, kurtosis and skewness values between + 1.5 and -1.5^20^, and + 2.0 and -2.0^21^ have been considered to indicate a normal distribution. The variables were normally distributed and parametric methods were used for data analysis.

## Results

 With randomization, it was controlled whether there was a difference between the patients assigned to the intervention and control groups in terms of descriptive characteristics. As a result of the analyses, it was found that the patients in the both groups were statistically similar in terms of descriptive characteristics** (***P* > 0.05) and that the distribution of the patients into the intervention and control groups was homogeneous (Table 1).

**Table 1:** Sociodemographic and Disease-Related Characteristics of the Intervention and Control Groups

**Variable**		**Intervention Group** **(** ***n*** **=30)**		**Control Group** **(** ***n*** **=30)**		**χ**^2^	***P***
		***n ***	**%**	***n ***	**%**		
Gender	Female	15	50.0	12	40.0	0.61	0.44
	Male	15	50.0	18	60.0		
Educational status	Primary school	23	76.7	20	66.7	1.02	0.80
	Secondary school	3	10.0	4	13.3		
	High school	2	6.7	4	13.3		
	Bachelor’s or higher	2	6.7	2	6.7		
Marital status	Single	2	6.7	3	10.0	0.22	0.64
	Married	28	93.3	27	90.0		
Employment status	Employed	2	6.7	5	16.7	1.46	0.23
	Unemployed	28	93.3	25	83.3		
Place of residence	Province	12	40.0	14	46.7	5.70	0.06
	District	9	30.0	14	46.7		
	Village	9	30.0	2	6.7		
Support status	Alone	1	3.3	1	3.3	2.07	0.36
	Family	29	96.7	27	90.0		
	Nursing home	0	0.0	2	6.7		
Economic status	Income less than expenses	22	73.3	15	50.0	3.90	0.14
	Income equal to expenses	6	20.0	13	43.3		
	Income more than expenses	2	6.7	2	6.7		
Diagnosis	Lung cancer	5	16.7	9	30.0	2.11	0.55
	Breast cancer	5	16.7	6	20.0		
	Gastrointestinal cancer	8	26.7	5	16.7		
	Other	12	40.0	10	33.3		
Presence of another chronic disease	Yes	13	43.3	10	33.3	0.64	0.43
	No	17	56.7	20	66.7		
		**Mean±SD**		**Mean±SD**		***t***	***P***
Age	Mean age	60.13 ± 10.77		61.07 ± 13.78		-0.29	0.77

 The post-test state anxiety scores were 31.20 (SD = 3.38) for the intervention group and 52.90 (SD = 7.16) for the control group. According to Levene’s test, the variances were equal (*P* = 0.129). According to the ANCOVA findings, the group variable significantly affected the post-test state anxiety scores when adjustment was made for pre-test state anxiety scores (F = 240.398, *P* < 0.001, η^2^ = 0.808). In addition, pre-test state anxiety scores affected post-test results (F = 7.925, *P* = 0.007, η^2^ = 0.122). The estimated mean score was 30.255 (95% CI: 28.208‒32.302) for the intervention group and 53.845 (95% CI: 51.798‒55.892) for the control group, and this difference was significant (*P* < 0.001). These findings demonstrate that the experimental intervention was effective and that the anxiety levels of the participants in the intervention group decreased significantly (Table 2).

**Table 2:** Descriptive Statistics and ANCOVA Results Regarding State Anxiety, Trait Anxiety, and Fatigue Scores of the Intervention and Control Groups

**State Anxiety**	**Pre-test (Mean)**	**Post-test (Sd)**	**Post-test (Mean)**	**Post-test (Sd)**	**Estimated Mean (SE)**	**95% Confidence Interval (Estimated Mean)**
Intervention	52.67	6.98	31.20	3.38	30.255 (1.022)	28.208‒32.302
Control	45.20	8.40	52.90	7.16	53.845 (1.022)	51.798‒55.892
	**Sum of Squares**	**df**	**Mean Square**	**F**	***P***	**Partial Eta Square**
Adjusted Model	7284.967	2	3642.483	130.261	0.000	0.820
Pre-test State Anxiety	221.617	1	221.617	7.925	0.007	0.122
Group	6722.221	1	6722.221	240.398	0.000	0.808
Error	1593.883	57	27.963			
**Trait anxiety**	**Pre-test (Mean)**	**Pre-test (Sd)**	**Post-test (Mean)**	**Post-test (Sd)**	**Estimated Mean (SE)**	**95% Confidence Interval (Estimated Mean)**
Intervention	49.30	5.46	31.50	2.50	29.253 (0.860)	27.531‒30.975
Control	39.77	8.19	47.40	7.10	49.647 (0.860)	47.925‒51.369
	**Sum of Squares**	**df**	**Mean Square**	**F**	***P***	**Partial Eta Square**
Adjusted Model	4416.840	2	2208.420	123.653	0.000	0.813
Pre-test Trait Anxiety	624.690	1	624.690	34.977	0.000	0.380
Group	4201.396	1	4201.396	235.243	0.000	0.805
Error	1018.010	57	17.860			
**Fatigue**	**Pre-test (Mean)**	**Pre-test (Sd)**	**Post-test (Mean)**	**Post-test (Sd)**	**Estimated Mean (SE)**	**95% Confidence Interval (Estimated Mean)**
Intervention	6.59	1.81	2.28	0.44	1.796 (0.256)	1.284‒2.308
Control	3.38	1.50	5.01	1.71	5.501 (0.256)	4.989‒6.013
	**Sum of Squares**	**df**	**Mean Square**	**F**	***P***	**Partial Eta Square**
Adjusted Model	126.714	2	63.357	47.935	0.000	0.627
Pre-test fatigue	14.648	1	14.648	11.082	0.002	0.163
Group	104.684	1	104.684	79.201	0.000	0.582
Error	75.339	57	1.322			

 The mean post-test trait anxiety score was 31.50 (SD = 2.50) for the intervention group and 47.40 (SD = 7.10) for the control group. According to Levene’s test, the variances were equal (p = 0.059). According to the ANCOVA findings, the group variable had a significant effect on the post-test trait anxiety scores (F = 235.243, *P* < 0.001, η^2^ = 0.805). In addition, pre-test trait anxiety scores were found to have an effect on post-test results (F = 34.977, *P* < 0.001, η^2^ = 0.380). The estimated mean score was 29.253 (95% CI: 27.531‒30.975) for the intervention group and 49.647 (95% CI: 47.925‒51.369) for the control group, and this difference was significant (*P* < 0.001). These findings indicated that the experimental intervention was effective and that the trait anxiety levels of the participants in the intervention group decreased significantly (Table 2).

 The mean post-test fatigue score was 2.28 (SD = 0.44) for the intervention group and 5.01 (SD = 1.71) for the control group. According to Levene’s test, the variances were equal (p = 0.088). According to ANCOVA results, the group variable significantly affected post-test fatigue scores (F = 79.201, *P* < 0.001, η^2^ = 0.582). In addition, pre-test scores affected post-test scores (F = 11.082, *P* = 0.002, η^2^ = 0.163). The estimated mean score was 1.796 (95% CI: 1.284 - 2.308) for the intervention group and 5.501 (95% CI: 4.989‒6.013) for the control group, and the difference was significant (*P* < 0.001). These findings showed that the experimental intervention was effective and that the fatigue levels of the intervention group participants decreased significantly (Table 2).

## Discussion

 In this study, the effect of music intervention on fatigue and anxiety in cancer patients receiving chemotherapy was studied. According to the findings obtained in the study, it was seen that music positively affected fatigue and anxiety levels.

 The results of our study on the level of fatigue strengthen the idea that listening to music during chemotherapy reduces the level of fatigue in cancer patients undergoing chemotherapy. Fatigue is one of the important symptoms that occurs in patients undergoing chemotherapy treatment. It can reach dimensions that can affect the patients’ daily life activities, quality of life and treatments. There have been many attempts to reduce the symptom of fatigue, and one of them is to play music during chemotherapy. When similar studies on the subject are examined, it is emphasized that music intervention is effective in reducing fatigue.^1,2,9,15,22^ Alcântara-Silva et al^23^ reported in their study on cancer patients undergoing radiotherapy that music therapy had an effect on fatigue symptoms. Although the treatments and music interventions applied are different, it can be concluded that music is also effective in reducing fatigue in cancer patients. With music intervention, it is possible that the focus of the patient moves away from the chemotherapy by distracting attention during the treatment, and the intensity and anxiety decreases with the relaxing effect of music. In addition, emotional support provided by music intervention may be an effective factor in reducing fatigue.

 The results of our study, showing that state and trait anxiety are lowered in the intervention group, strengthen the idea that listening to music during chemotherapy in cancer patients undergoing chemotherapy reduces anxiety in patients. Similar to our study, Bro et al,^24^ who examined the effect of music intervention on anxiety in cancer patients in their randomized controlled study, reported a significant decrease in the anxiety level of the intervention group among lymphoma patients. In another randomized controlled study conducted by Burns et al,^22^ different music interventions were applied and it was found that the distress scores of the music listening group were lower. In many similar studies on the subject, it was emphasized that music intervention was effective on anxiety, and listening to music in patients undergoing chemotherapy reduced anxiety.^12,15,16,25-28^ It is thought that music intervention directly reduces people’s anxiety and physiological arousal and improves patients’ feelings of well-being and control.^26^ In addition, waiting for the chemotherapy cycle or starting the treatment may be worrisome. At this point, the relaxing effect of music intervention and the patient’s distraction to another direction, as well as providing emotional support to the patient with music which is a supportive method, may play a role in reducing anxiety. In the light of all these research results, it can be said that music can be applied as an alternative in reducing fatigue and anxiety and coping with these symptoms. Moreover, its low cost and being a noninvasive procedure can provide convenience and widespread use in practice. In addition, considering that music intervention is not widely applied in Turkey, based on the results of the research, we can say that oncology nurses can include music intervention in their nursing care for patients undergoing chemotherapy.

## Limitations

 The research was carried out at a single center, so the generalizability of the research may be limited. Another limitation is that the research was not conducted on a single cancer diagnosis due to the small number of patients. In addition, although single blinding was performed and the participants did not know to which group they were assigned during the randomization process, double blinding could not be performed since the intervention and interview were performed by the same researcher.

## Conclusion

 The study shows that music intervention can be used in nursing practices for cancer patients during chemotherapy. Its low cost and non-invasive nature also provide ease of application. Therefore, we can recommend the application of music intervention in outpatient chemotherapy units. Considering that the research period was short, it may be suggested to investigate the long-term effects of music interventions in larger samples.
